# Prevalence of non-alcoholic fatty liver disease in four different weight related patient groups: association with small bowel length and risk factors

**DOI:** 10.1186/s13104-015-1224-7

**Published:** 2015-07-03

**Authors:** Andreas Hillenbrand, Brigitte Kiebler, Cornelia Schwab, Ludger Scheja, Pengfei Xu, Doris Henne-Bruns, Anna Maria Wolf, Uwe Knippschild

**Affiliations:** Department of General and Visceral Surgery, Ulm University Hospital, Albert-Einstein-Allee 23, 89081 Ulm, Germany; Department of Pathology, Ulm University Hospital, Albert-Einstein-Allee 23, 89081 Ulm, Germany; Department of Biochemistry and Molecular Cell Biology, University Medical Center Hamburg-Eppendorf, 20246 Hamburg, Germany

**Keywords:** NASH, Non-alcoholic fatty liver disease, Morbidly obese, Small bowel length

## Abstract

**Background:**

Non-alcoholic steatohepatitis (NASH) is an obesity associated common cause of liver inflammation and there are concerns that it may turn out to be the most common cause of liver failure as prevalence of obesity increases. We determined the prevalence of NASH in relation to gender and body mass index (BMI). Furthermore, we assessed the association of NASH with the length of the small bowel.

**Methods:**

124 liver samples obtained during routine operations were examined looking for NAFLD Activity Score (nonalcoholic fatty liver disease). The length of small bowel was measured intraoperatively. For evaluation, patients were divided into four groups according to their BMI (group 1: normal weight, group 2: overweight, group 3: grade I/II morbidly obese, and group 4 grade III morbidly obese patients).

**Results:**

BMI showed a strong positive correlation with risk of NASH and a weak positive correlation with small bowel length. No normal weight patient was at risk of NASH, whereas in group 2 14% had uncertain and 32% definite NASH. In group 3 11% had uncertain and 27% definite NASH. In group 4 nearly two-thirds were classified as uncertain or definite NASH. Median length of small bowel in all patients was 450 cm (range 226–860 cm). Within group 4, patients with definite/uncertain NASH had a longer small bowel than patients without NASH.

**Conclusions:**

Prevalence of NASH is high in morbidly obese. Small bowel length could influence the complex etiology of the disease.

## Background

Non-alcoholic fatty liver disease (NAFLD), defined by non-alcohol related excessive fat accumulation in the liver, is a leading cause of chronic liver disease worldwide. Obesity is a major risk factor for NAFLD and NAFLD is often linked to other obesity related diseases such as insulin resistance and several features of metabolic syndrome. Although simple steatosis carries a relatively benign prognosis, a significant proportion of patients will progress to non-alcoholic steatohepatitis (NASH) [1]. NASH is the advanced form of NAFLD, leading to liver cirrhosis, end-stage liver disease, and hepatocellular carcinoma [2]. As the worldwide obesity epidemic continues to increase, NASH will surpass chronic hepatitis C infection as the primary indication for orthotopic liver transplantation [3]. Although the pathogenesis of NAFLD/NASH is not yet fully understood, excess intracellular fatty acids, oxidative stress, inflammatory cytokines/adipocytokines, and mitochondrial dysfunction in association with a genetic predisposition seem to contribute to progression from steatosis to more advanced liver inflammation and fibrosis [4]. Insulin resistance also is reported to be a risk factor for the presence of NASH, irrespective of obesity [5].

Histologically, the presence of a few steatotic hepatocytes in the liver is considered to be normal. The defining threshold for NALFD is an involvement of more than 5–10% of the liver. Steatosis of hepatocytes in NAFLD typically affects perivenular regions of the liver. In NASH, steatosis is accompanied by intralobular inflammation and hepatocyte injury, usually in the form of ballooning. Ongoing hepatic damage in NASH can lead to fibrosis and cirrhosis in some cases [6]. However, which conditions drive the progress of NAFLD towards NASH is not yet fully understood. As liver biopsy is mandatory for diagnosis of NASH, larger epidemiologic studies are challenging.

In this retrospective study, we analyzed 136 liver samples obtained from patients operated upon in our surgical department and determined the prevalence of NASH in relation to gender and body mass index (BMI). Furthermore, we assessed the association of NASH with fasting insulin levels, hepatic transaminases as well as the length of the small bowel measured intraoperatively.

## Methods

### Patients

Liver samples were obtained from 136 patients (69 males; 67 females) operated upon in the Department of General and Visceral Surgery at the University Hospital Ulm between 2003 and 2007. The study was performed with the permission of the independent local ethics committee of the University of Ulm (approval 73/2009). A written consent was obtained from patients prior to inclusion in this study. Patients with a history of alcohol abuse [more than 40 g (males)/20 g (females) per day] were excluded. 12 out of these 136 patients were excluded since a non-NASH liver disease was diagnosed (e.g., mainly intrahepatic cholestasis followed by liver metastasis and viral hepatitis/biliary cirrhosis). As shown in Table 1, out of the 124 remaining patients (100%), 71 patients (57%) were operated upon for malignant disease, mainly colorectal carcinoma, 19 patients (15%) for benign disease, mainly pancreatitis and diverticulitis of sigmoid colon, and 34 patients (27%) received a bariatric procedure. 61 patients were females (49%) and 63 patients were males (51%). 65% of male patients suffered from a malignant disease (mostly colorectal carcinoma, followed by pancreatic carcinoma and esophageal/gastric cancer). On 19% of the male patients a bariatric procedure was performed, and 16% of male patients were operated on for benign disease, predominantly benign pancreatic disease followed by benign small or large bowel disease. 49% of female patients were operated on for malignant disease (mostly colorectal carcinoma followed by pancreatic carcinoma and esophageal/gastric cancer. 36% of female patients underwent a bariatric procedure and 15% of female patients were operated on for benign disease, predominantly benign changes of the large bowel followed by a benign pancreatic tumor.

**Table 1:** Classification of patients according to their BMI (group 1: normal weight; group 2: overweight; group 3: I/II° obesity; group 4: III° obesity)

Group	BMI (kg/m^2^)	Malignant disease	Benign disease	Morbid obesity
All patients (n = 124; ♀61, ♂63)		n = 71; ♀30, ♂41	n = 19; ♀9, ♂10	n = 34; ♀22, ♂12
1 (n = 32; ♀12, ♂20)	18.5–24.9	n = 25; ♀10, ♂15	n = 7; ♀2, ♂5	n = 0; ♀0, ♂0
2 (n = 36; ♀13, ♂23)	25.0–29.9	n = 30; ♀11, ♂19	n = 6; ♀2, ♂4	n = 0; ♀0, ♂0
3 (n = 22; ♀12, ♂10)	30.0–39.9	n = 14; ♀7, ♂7	n = 6; ♀5, ♂1	n = 2; ♀0, ♂2
4 (n = 34; ♀24, ♂10)	≤40	n = 2; ♀2, ♂0	n = 0; ♀0, ♂0	n = 32; ♀22, ♂10

Patients were between 21 and 90 years old (median of all patients 59 years; female patients 23–86 years, with a median of 55 years; male patients 21–90 years, with a median of 62 years). Liver samples were taken from the left liver lobe, and shock frozen in liquid nitrogen or fixed in formalin. Serum levels of alanine transaminase (ALT) and fasting insulin were obtained. The length of small bowel was accurately measured intraoperatively from the ligament of Treitz to the ileocecal junction strictly on the mesenteric edge.

For evaluation, patients were divided into four groups according to their BMI (Table 1). Normal weight patients represent group 1, overweight patients constitute group 2, grade I/II morbidly obese patients are in group 3, and grade III morbidly obese patients are in group 4. Patients in group 4 were mainly operated upon for morbid obesity (31 out of 34 patients in this group). Patients operated upon for benign diseases had similar BMI values compared to patients operated on for malignant diseases (median 27.5 kg/m^2^; range 17.6–38.0 vs. median 26.1 kg/m^2^; range 18.9–42).

Blood samples were taken 1 day prior to operation in a fasting state at 8 am. The study was approved by the local ethics committee (study number 112/2003).

### Histological staining

After macroscopic assessment (size, color, consistency), the resected liver specimens were fixated in 10% neutral buffered formalin for 12 h and subsequently dehydrated by passing the tissue through a series of increasing alcohol concentrations. After dehydration, the tissues were embedded in paraffin and trimmed to 3 µm sections. After deparaffinization and rehydration with decreasing strengths of alcohol, sections were stained with Mayer’s hematoxylin (Chroma, Münster) for 15 min, then washed in running tap water for 20 min followed by counterstaining with eosin (Chroma, Münster). After dehydration in 95% and absolute alcohols, sections were cleared in xylene (Merck, Darmstadt) and mounted in Permount (Thermo Fisher Scientific Inc).

### NAFLD Activity Score (NAS)

Sections were analyzed at 200× magnification with the semi-quantitative scoring system according to Kleiner et al. [7]. Table 2 by an expert hepato-pathologist. The NAFLD Activity Score (0–8 points) is the sum of scoring for steatosis (0–3 points), inflammation (0–3 points) and ballooning (0–2 points). A score of at least 5 is defined as NASH.

**Table 2:** NAFLD activity scoring system according to Kleiner et al

NAFLD Activity Score (NAS) (0–8)		
Sum of scores for steatosis, lobular inflammation and hepatocellular ballooning		
≥5 probable or definite NASH		
3–4 uncertain NASH		
≤2 no NASH		
Steatosis (0–3)	Lobular inflammation (0–3)	Hepatocyte ballooning (0–2)
0: 5% hepatocytes involved	0: none	0: none
1: 5–33% of hepatocytes involved	1: <2 foci per × 200 field	1: few ballooned cells
2: 33–66% hepatocytes involved	2: 2–4 foci per × 200 field	2: many cells/prominent ballooning
3: >66% hepatocytes involved	3: >4 foci per × 200 field	
Correlation between total NAFLD activity scores and an overall histological diagnosis of steatohepatitis		
NAFLD activity score and histological diagnosis of steatohepatitis		
≥5 probable or definite NASH		
3–4 uncertain NASH		
≤2 no NASH		

### Statistical analysis

All values were expressed as arithmetic mean, standard deviation, median, and range. Statistical analysis was performed using Winstat software for windows (R. Fitch Software Version 2009.1). Data were statistically analyzed using the Kruskal–Wallis test (KW) for differences within the collective. If significant differences were found, gender-specific analysis was performed (KW test). To test differences between two groups, the Mann–Whitney U test (MWU) was used. Statistical significance was tested at p < 0.05. Boxplots were chosen to present results, whereby top and bottom of the rectangle represent the 25th and 75th percentile and the line within the rectangle represents the median. The whiskers extend from the 5th percentile to the 95th percentile. Outliners are marked with a circle. Correlations between the different subgroups were statistically analyzed using non-parametric Spearman’s correlation test, whereby correlation coefficients are indicated by r. Correlation coefficient values between 0.3 and 0.7 (−0.3 and −0.7) indicate a moderate, values between 0.7 and 1.0 (−0.7 and −1.0) indicate a strong positive (negative) linear relationship. Statistical significance was tested at p < 0.05 and a tendency at 0.05 < p < 0.10. No adjustments were made for multiple statistical comparisons.

## Results

### Liver and steatosis in the BMI groups

**Figure 1:**
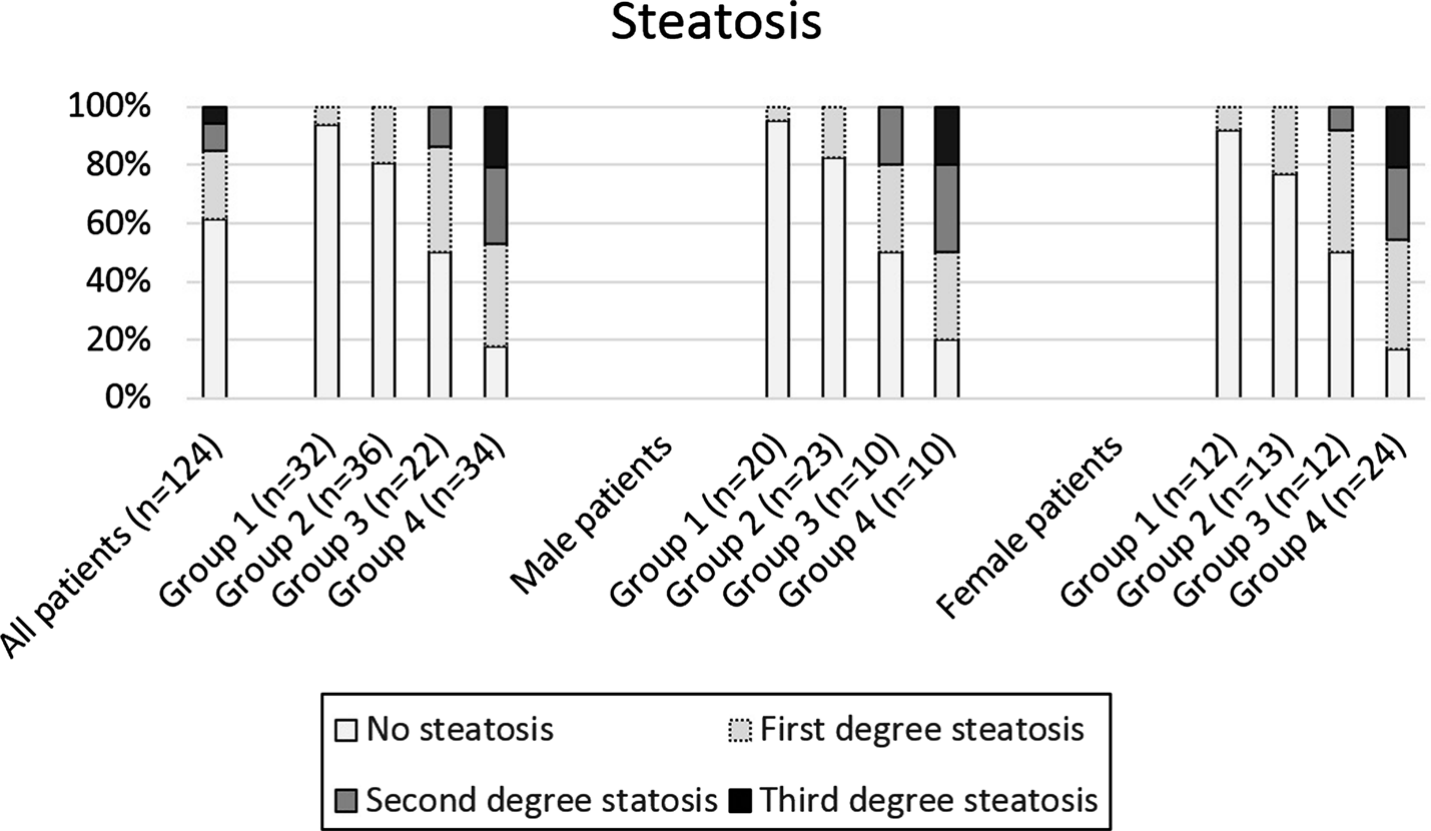
Hepatic steatosis in the four BMI groups (group 1: BMI 18.5–24.9 kg/m^2^, group 2: 25.0–29.9 kg/m^2^, group 3: 30.0–39.9 kg/m^2^ and group 4: ≥40 kg/m^2^) was determined with H&E staining. Steatosis was evaluated semi-quantitatively described by Kleiner et al. [7]. No steatosis: 0–5% of parenchymal involvement by steatosis, first degree steatosis: 5–33% of parenchymal involvement by steatosis, second degree steatosis: 33–66% of parenchymal involvement by steatosis, and third degree steatosis: >66% of parenchymal involvement by steatosis.

Steatosis was estimated by histology after staining. Median fat content of all patients was 4% (female patients: median 4%, range 0–90%; male patients: median 2%, range 0–80%). Overall, 76 patients (61%) had no or low grade fatty degeneration of the liver (<5%), grade 1 liver steatosis (5–33%) was found in 29 patients (23%), grade 2 liver steatosis (34–66%) in 12 patients (10%), grade 3 liver steatosis (>66%) in 7 patients (6%). As showed in Figure 1, group 1 patients had a median fat content of 1% (range 0–20%), group 2 patients of 3% (range 0–30%), group 3 patients of 10% (range 0–60%), and group 4 patients of 30% (range 1–90%). There was no gender-specific difference in fat content of the liver. As expected, BMI showed a strong positive correlation with fat content of the liver (correlation coefficient r = 0.70; p < 0.01).

### Lobular inflammation in the BMI groups

**Figure 2:**
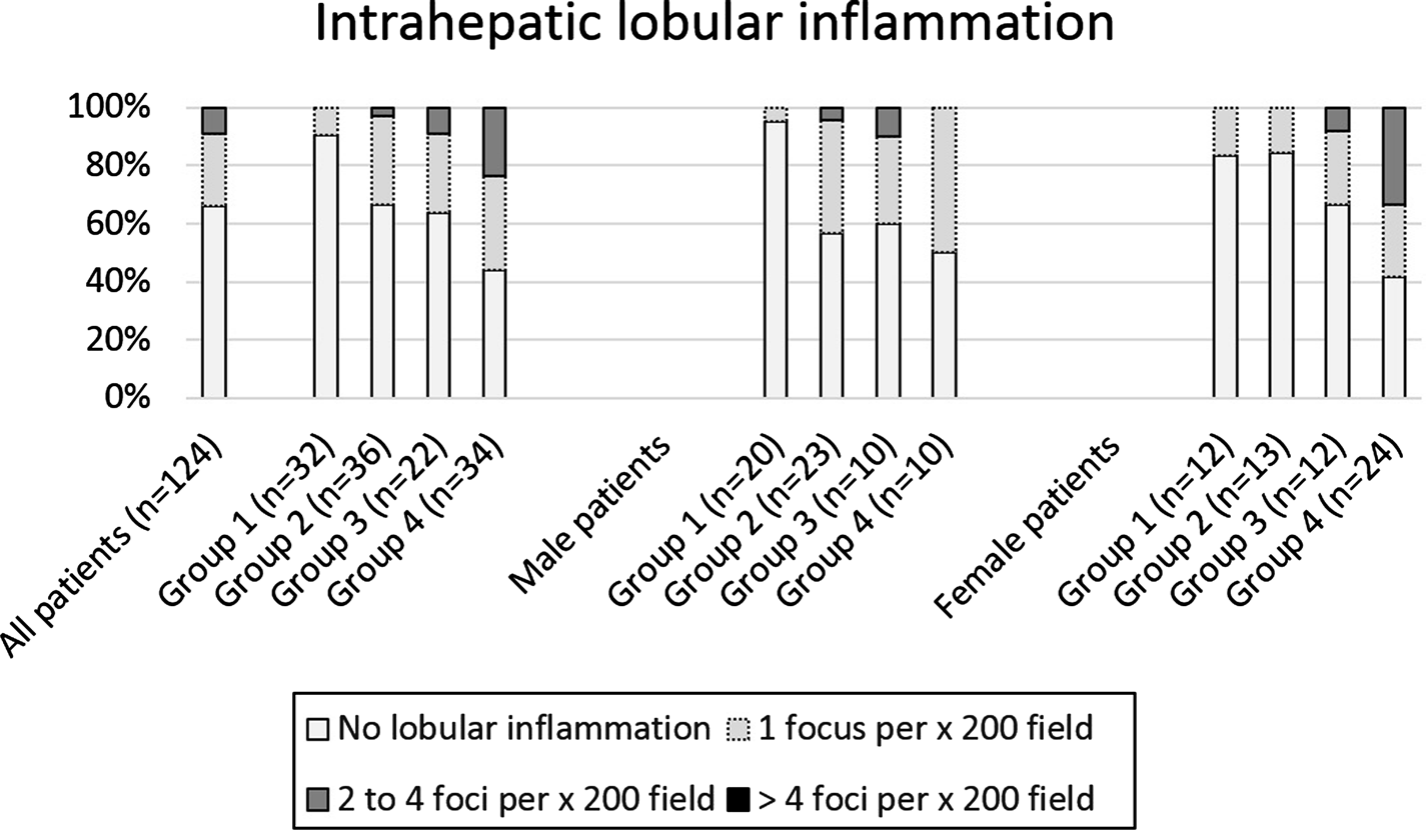
Intrahepatic lobular inflammation was determined in the four BMI groups (group 1: BMI 18.5–24.9 kg/m^2^, group 2: 25.0–29.9 kg/m^2^, group 3: 30.0–39.9 kg/m^2^ and group 4: ≥40 kg/m^2^) with H&E staining (number of foci of lobular inflammation per × 200 field). No lobular inflammation was detectable in 81 out of 123 patients (65.9%), 31 patients showed <2 foci of lobular inflammation per × 200 field (25.2%), and 11 patients showed between 2 and 4 foci (8.9%). No patient had more than 4 foci of lobular inflammation per × 200 field.

Lobular inflammation was scored by determining the number of foci of lobular inflammation per × 200 field. Inflammatory foci are aggregates of a mixed population of leukocytes usually containing a significant number of neutrophils. No lobular inflammation was detectable in 82 out of 124 patients (66%). 31 patients showed <2 foci of lobular inflammation per × 200 field (25%), whereas 11 patients showed a lobular inflammation between 2 and 4 foci (9%). The highest inflammation score, i.e., >4 foci of lobular inflammation per × 200 field was not observed in any patient. Overall, lobular inflammation tended to increase with BMI, as shown by the percentage of patients exhibiting detectable inflammation (group 1: 9%; group 2: 33%; group 3: 36%; group 4: 56%; Figure 2).

### Hepatocyte ballooning in the BMI groups

**Figure 3:**
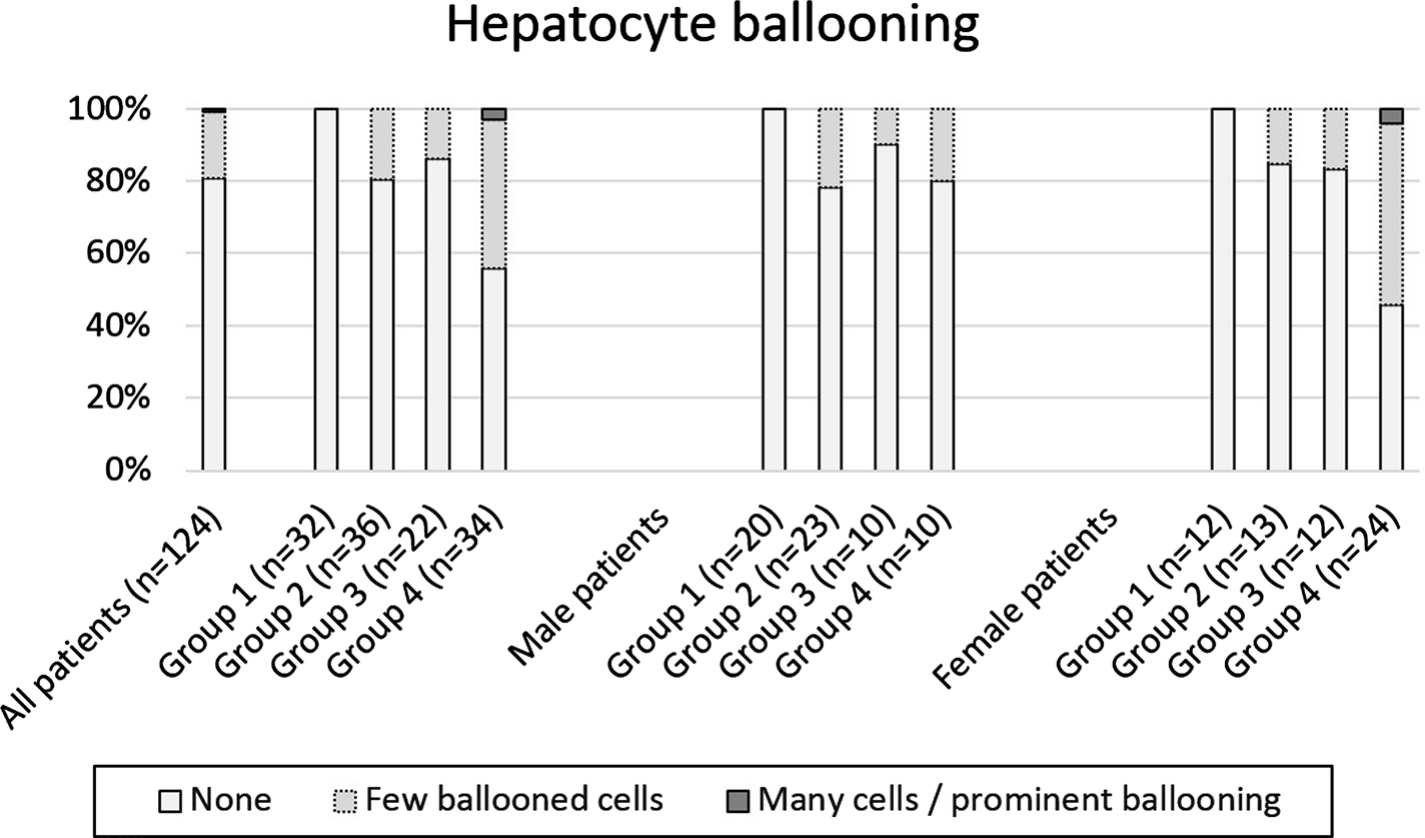
Hepatocyte ballooning was determined with H&E staining in the four BMI groups (group 1: BMI 18.5–24.9 kg/m^2^, group 2: 25.0–29.9 kg/m^2^, group 3: 30.0–39.9 kg/m^2^ and group 4: ≥40 kg/m^2^). No hepatocyte ballooning was seen in 100 out of 124 patients (81%) and in all 32 group 1 patients. 14% each of group 2 and 3 patients had a few ballooned cells. One patient of group 4 had many/prominent ballooned cells and 14 patients (41%) a few ballooned cells.

Ballooned liver cells are markedly enlarged and display a rounded shape with a weakly stained, reticulated cytoplasm. Hepatocyte ballooning is the result of severe cell injury, finally leading to cell death. Overall 24 out 124 patients (19%) had signs of hepatocyte ballooning (Figure 3). No hepatocyte ballooning was detected in group 1 patients. 14% of group 2 and group 3 patients had few ballooned cells. No patient in group 2 and group 3 had many or prominent ballooned cells. In group 4, 1 patient (3%) had many/prominent ballooned cells and 14 patients (41%) few ballooned cells.

### NAFLD Activity Score (NAS)

**Figure 4:**
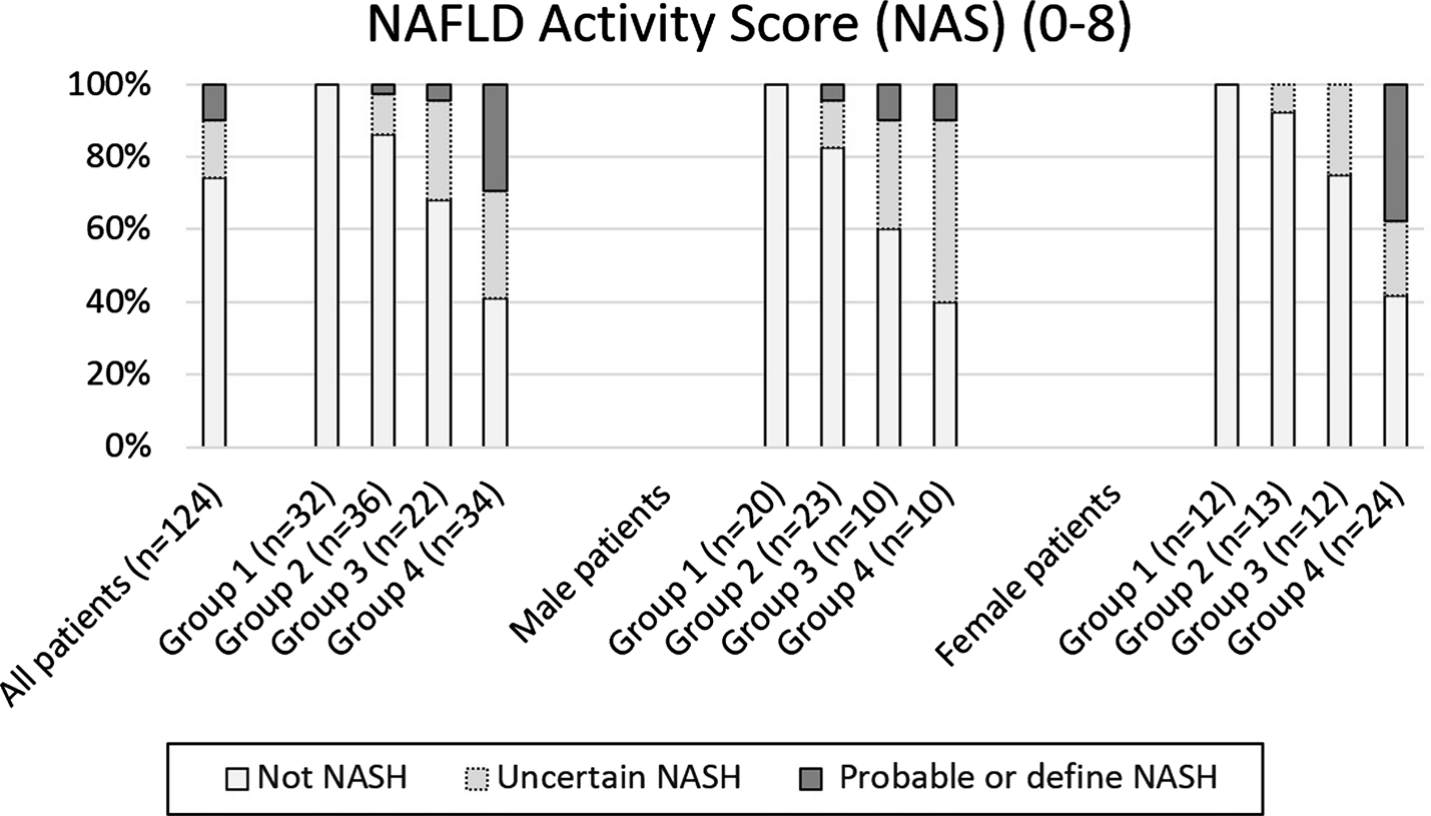
NAFLD Activity Score according to the scoring system of Kleiner et al. 12 patients (10%) had probable or definite NASH and a further 20 patients (16%) had uncertain NASH. In group 1, no patient was at risk of NASH, in groups 2 and 3, 1 patient each (3% and 5%, respectively) had probable or definite NASH and a further 4 and 6 patients (11% and 27%, respectively) had uncertain NASH. In group 4, 10 patients (29%) were classified as uncertain NASH and a further 10 (29%) as definite NASH.

**Figure 5:**
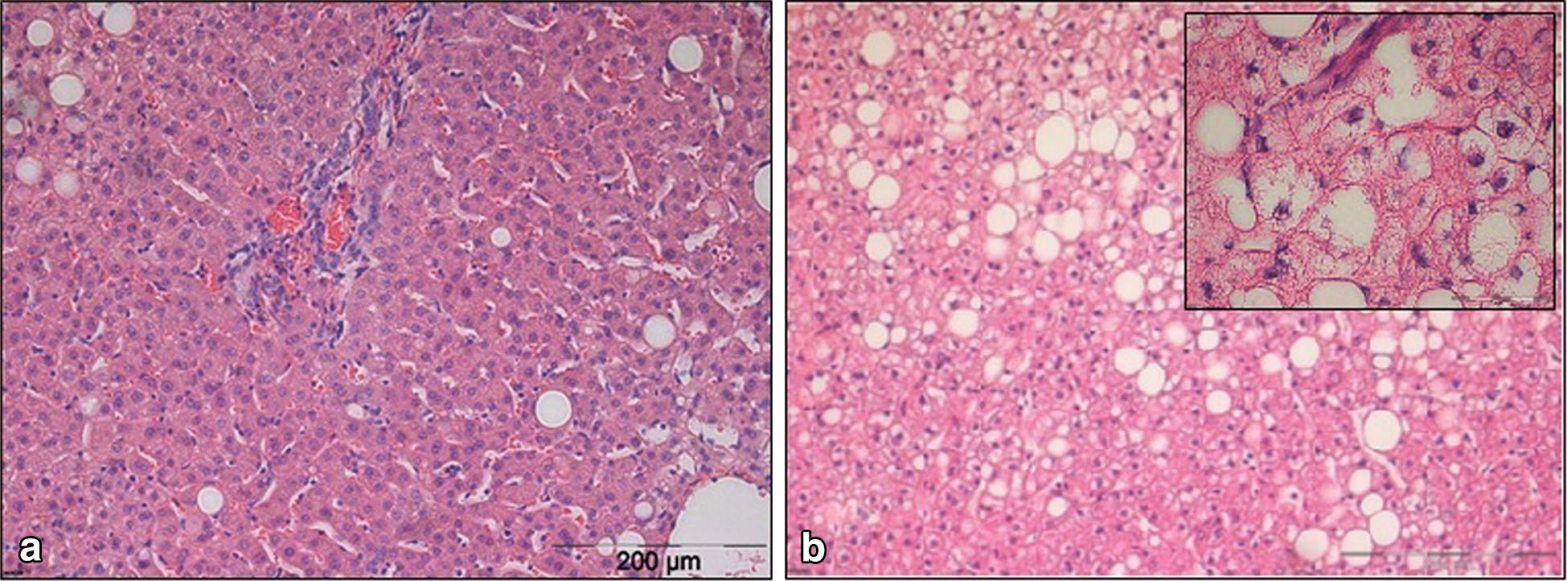
HE staining of liver samples (magnification 150×). **a** Liver tissue of a 49 years old patient operated for malignant disease (BMI 28.4 kg/m^2^) with a low grade fatty degeneration of the liver (<5%) and no lobular inflammation or hepatocyte ballooning. **b** Liver tissue of a 52 years old patient operated for morbid obesity (BMI 60.0 kg/m^2^) with grade 2 liver steatosis (40%) and some lobular inflammation and hepatocyte ballooning (*top right* an enlarged view; magnification 600×).

According to the scoring system by Kleiner et al. [6], 12 patients (10%) had probable or definite NASH while a further 20 patients (16%) had uncertain NASH (Figure 4). In group 1, no patient was at risk of NASH, in groups 2 and 3, 1 patient each (3 and 5%, respectively) had probable or definite NASH and a further 4 and 6 patients, respectively (11 and 27%) had uncertain NASH. In group 4, 10 patients (29%) were classified as uncertain NASH and a further 10 (29%) as definite NASH. In this group, 40% (4 out of 10) of male patients and 42% (10 out of 24) of female patients had no NASH. With regard to gender, 50% of males in group 4 (5 out of 10) were classified as uncertain and 10% (1 out of 10) were classified as probable or definite NASH, while 21% (5 out of 24) of group 4 females had uncertain NASH and 38% (9 out of 24) definite NASH. Group 4 patients classified as probable or definite NASH had a similar BMI compared to no NASH group 4 patients (median 52.8 kg/m^2^, range 40.8–66.5 kg/m^2^ vs. median 54.3 kg/m^2^, range 35.0–66.4 kg/m^2^). Figure 5 shows HE staining of two liver samples.

### Small bowel length, insulin levels, triglycerides, and transaminases

Intraoperative small bowel length measurement was documented in 108 patients. As shown in Table 3, median length of small bowel in all patients was 450 cm (range 226–860 cm) and small bowel length showed a weak positive correlation with BMI (r = 0.30; Kruskal–Wallis test for different small bowel length between the four BMI subgroups: p < 0.01). Patients with definite/uncertain NASH had a longer small bowel than patients without NASH (p < 0.01). The small bowel of group 4 patients was significant longer than of patients in groups 1–3 (p < 0.01). Within group 4, patients with definite/uncertain NASH had a significantly longer small bowel than group 4 patients without NASH (p = 0.04). Group 2 and 3 patients with definite/uncertain NASH did not have a significantly longer small bowel than group 2/3 patients without NASH (p = 0.24).

**Table 3:** Small bowel length

	Median (cm)	Range (cm)
All patients	450	226–860
Patients without NASH	440	(226–770)
Patients with definite/uncertain NASH	520	(240–860)
Groups 1–3 patients	430	(226–860)
Group 4 patients	495	(312–760)
Group 2/3 patients without NASH	430	226–630
Group 2/3 patients with definite/uncertain NASH	455	240–860
Group 4 patients without NASH	555	312–760
Group 4 patients with definite/uncertain NASH	480	360–520

We analyzed fasting insulin levels in 31 group 4 patients. Patients with definite/uncertain NASH had higher (not significant) insulin levels (median 31.4 mU/l; range 8.9–47.3 mU/l; normal range 2.6–24.9 mU/l) than group 4 patients without NASH (median 19.8 mU/l; range 4.8–40.0 mU/l; p = 0.065). Group 2/3 patients (definite/uncertain NASH vs. no NASH) showed no difference in fasting insulin levels (data not shown). We also measured triglyceride (TG) levels, because macrovesicular steatosis occurs as a manifestation of excessive TG accumulation in the liver. We found a weak positive correlation between TG levels and BMI (r = 0.26), but within a BMI group, patients with definite/uncertain NASH had only slightly and not significantly higher TG levels than patients without NASH (group 3: median TG levels in patients without NASH: 138 mg/dl; range 52–364 mg/dl, median TG levels in patients with definite/uncertain NASH: 170 mg/dl; range 99–272 mg/dl, p = 0.78; group 4: median 111 mg/dl; range 83–210 mg/dl vs. median 148 mg/dl; range 71–223 mg/dl, p = 0.16).

Regarding hepatic enzymes, we found a moderate positive correlation between ALT levels and BMI (r = 0.34). Patients with definite/uncertain NASH had significantly higher ALT levels (median 50.5 U/l; range 16.0–116.0 U/l; normal range: male <45 U/l, female <35 U/l) than patients without NASH (median 25.5 U/l; range 12.0–433.0 U/l; p < 0.01). Within a BMI group, we found different results in BMI groups 3 and 4 patients. BMI group 3 patients with definite/uncertain NASH had only slightly, but not significantly, higher ALT levels than patients without NASH (median ALT levels in patients without NASH: 28 U/l; range 13–433 U/l, median ALT levels in patients with definite/uncertain NASH: 76 U/l; range 18–116 U/l, p = 0.07) while BMI group 4 patients with definite/uncertain NASH had significantly higher ALT levels than BMI group 4 patients without NASH (median ALT levels in patients without NASH: 28 U/l; range 14–38 U/l; median ALT levels in patients with definite/uncertain NASH: 46 U/l; range 20–114 U/l, p < 0.01).

## Discussion

As the worldwide obesity pandemic grows, rates of metabolic syndrome and its hepatic manifestation, NAFLD, rise as well. The true prevalence of NAFLD and NASH is unknown and disease definition and modalities used for diagnosis and epidemiology studies are not standardized [8]. Based on imaging studies, the prevalence of NAFLD in adult populations ranges between 14 and 31% and in the obese, incidence of steatosis increases [9]. In obese individuals (BMI > 30 kg/m^2^), evidence of liver steatosis was found in up to 91% on ultrasound [10]. These results are comparable with our analysis with a prevalence of 70% steatosis in obese patients. Hepatic steatosis without NASH is a benign condition that does not impair liver function or lead to organ damage [11]. However, when hepatic cell necrosis and inflammation appear, fatty liver disease can progress into NASH and ultimately lead to cirrhosis, end-stage liver disease and hepatocellular carcinoma.

Data on prevalence of NASH are more difficult to assess than of simple steatosis, since a liver biopsy would be required to reliably diagnose NASH. NASH is estimated to occur in about 2–3% of the general population while it is found in 25–36% in the morbidly obese [10, 12]. We analyzed the prevalence of NASH in 124 patients, subdivided into four different groups depending on patient BMI. We did not find NASH in normal weight patients (BMI < 25 kg/m^2^). In 36 overweight patients (BMI 25 to <30 kg/m^2^) one patient was staged as probable or definite NASH, and four patients were staged as uncertain NASH according to the NASH scoring system by Kleiner et al. [7]. With increasing body weight, there is an increased prevalence of NASH: 6 out of 22 patients with first or second degree obesity (BMI 30 to <40 kg/m^2^) and 20 out of 34 patients with third degree obesity (BMI ≥ 40 kg/m^2^) were staged as uncertain NASH or as probable or definite NASH. Gender seems to matter in that male patients display greater tendency to NASH than female patients [13]. In this study, we found no gender-specific difference in prevalence of NASH. However one should remember that patients in group 4 (BMI > 40 kg/m^2^) were almost 20 years younger than patients in groups 1–3. Since NAFLD and NASH are commonly diagnosed in morbidly obese patients, a liver biopsy is now recommended in bariatric surgery [14].

The hypothesis that the length of the small intestine is related to obesity has been under discussion for a long time. Medical literature contains limited and often incomprehensible information regarding small bowel length in living patients. Average length is mostly given as between 280 cm and approximately 600 cm (ranges from 260 to 800 cm) [15–17], but average length can vary up to almost 10 m [18]. The method employed for measurement and the conditions under which the measurements were made could explain the different results (autopsy studies, double balloon endoscopy, swallowed polyvinyl tube, intraoperative measurement, and here measurement along the mesenteric/antimesenteric border and open/laparoscopic approach). Additionally, there are conflicting data regarding small bowel length and its relationship to height and weight. While existing data confirm a relationship between body weight and length of small bowel [19], Nassif et al. found no relationship in overweight patients submitted to bariatric surgery [20]. Remarkably, we found a positive correlation between small bowel length and patient BMI. Furthermore, the small bowel of NASH patients was significantly longer than the small bowel of patients without NASH. It is problematic that a total of six different surgeons measured the small bowel intraoperatively, since different applied degrees of tension could modify the results. Small bowel measurement and bariatric procedures for all patients with morbid obesity (in group 4: 32 out of 34 patients) were conducted by one surgeon only. All procedures were executed in an open approach. The outcomes of future research may determine whether bariatric surgery will be one option for treatment of the most progressive type of NAFLD [21].

Consistent with other studies, we found ALT levels were related to advanced liver disease [22, 23], however only in BMI group 4 patients. BMI group 3 patients with and without NASH showed no significant difference in ALT levels. However, we found no significantly higher TG levels in NASH patients than patients without NASH within the same BMI group. Insulin resistance is believed to be a central mechanism involved in the development of hepatic steatosis. We found higher fasting insulin levels in obese patients (BMI > 30 kg/m^2^) with definite/uncertain NASH than in morbidly obese patients (BMI > 40 kg/m^2^) without NASH. However we found no difference in fasting insulin levels in overweight and morbidly obese patients with a BMI below 40 kg/m^2^. Current therapy for NASH has focused on improving insulin resistance and mediators of inflammation, factors probably associated with disease progression. Insulin resistance can be targeted through a multifaceted approach involving weight loss, surgical intervention, or pharmacological therapy [8]. In the morbidly obese, the weight loss approach with the most durable effect on obesity and NASH is bariatric surgery, which can achieve, in particular with a malabsorptive procedure, an improvement of NAFLD both biochemically and histologically [24].

Our study has several strengths and limitations. All patients were recruited and operated on in the same hospital. Blood samples of all groups were collected via the same protocol, simultaneously stored, and processed with the same assay. The main limitation of our study is its cross-sectional design, so we cannot conclude that clinical markers predict progression of liver disease, as this would imply examining these components prospectively in the same individuals. Further limitations relate to the fact that many group 4 patients underwent medical weight loss strategies prior to presenting for surgery and the prevalence of medications known to impact NASH and metabolism could not be accounted for.

## Conclusion

Simple steatosis of the liver is highly prevalent especially in the obese. In our study, we found steatosis of the liver in more than 80% of morbidly obese patients, however, probable/definite NASH was found in only 30% of these patients (and a further 30% of these patients were classified as uncertain NASH). The exact pathogenesis and natural history are still being defined. Therapy currently addresses the features of metabolic syndrome, including diabetes, obesity, and dyslipidemia, however, if a connection between NASH and small bowel length is confirmed, malabsorptive bariatric surgery should be considered as a further option.

## Endnote

This work is dedicated to the memory of Dr. Cornelia Schwab, who died on May 07, 2011.

## Abbreviations

NASH: non-alcoholic steatohepatitis
BMI: body mass index
NAFLD: non-alcoholic fatty liver disease
ALT: alanine transaminase
KW: Kruskal–Wallis test
MWU: Whitney U test
NAS: NAFLD Activity Score
TG: triglyceride
